# Organic Pollutant Penetration through Fruit Polyester Skin: A Modified Three-compartment Diffusion Model

**DOI:** 10.1038/srep23554

**Published:** 2016-03-24

**Authors:** Yungui Li, Qingqing Li, Baoliang Chen

**Affiliations:** 1 Department of Environmental Science, Zhejiang University, Hangzhou 310058, China; 2 key Laboratory of Solid Waste Treatment and Resource Recycle, Ministry of Education, Southwest University of Science and Technology, Mianyang 621010, China; 3 Zhejiang Provincial Key Laboratory of Organic Pollution Process and Control, Hangzhou 310058, China

## Abstract

The surface of plants is covered by a continuous but heterogeneous cuticular membrane (CM). Serving as the first protective barrier, the uptake and transport behavior of organic pollutants at this interface continue to engage the research efforts of environmental chemist. To date, the contributions of cuticular components as a defense against the organic pollutants penetration remain unresolved. In this study, the unsteady-state penetration characteristics of phenanthrene (PHE) through isolated fruit CM was investigated. PHE penetration was differentiated by three cuticular compartments: epicuticular waxes (EW), cuticle proper (CP) and cuticular layer (CL). The driving force for PHE penetration was ascribed to the sharp concentration gradient built up endogenously by cuticular compartments with different lipophilic affinities. A modified penetration model was established and verified in terms of its general suitability for the hydrophobic chemicals and CMs of various plant species (apple, tomato and potato). The new three-compartment model demonstrates much higher accuracy in characterizing the uptake and transport behavior of semivolatile chemicals with fewer limitations in terms of environmental conditions and complexity (e.g., coexisting contaminants and temperature). This model could contribute to a more comprehensive understanding on the role of polymeric lipids in the organic pollutant sorption and transport into plants.

The penetration and accumulation of organic pollutants in plants have crucial impacts on their global cycling, bio-availability and then food safety in the environment^1^,^2^. It is widely recognized that organic pollutants primarily accumulate in the epidermis of plants^3^,^4^. Plant surfaces are covered by cuticle, a continuous but heterogeneous membrane comprising waxes, cutin, polysaccharides and, in some plant species, cutan^5^,^6^,^7^. Waxes can be classified into a distinctly outmost amorphous layer called epicuticular waxes (EW) and highly ordered crystalline structures embedded in the cuticular matrix called intracuticular waxes. Intracuticular waxes and cutin constitute the cuticle proper (CP). Cutan and polysaccharides wrapped outside are known as the cuticular layer (CL)^8^,^9^,^10^. Keyte *et al*.^11^ found that the penetration of phenanthrene (PHE), a typical persistent organic pollutant, was much faster through the surface of moss (which has no typical cuticle) than through that of a spinach leaf (which has a typical cuticle). The cuticle is an essential barrier that hinders the penetration of organic pollutants into the plant, but the contributions of cuticular components to such penetration remain unresolved.

Numerous studies have explored the penetration characteristics and diffusion behaviors of organic pollutants in plant cuticles. The penetration of organic compounds through plant cuticles is recognized as the rate-limiting step of pollutant uptake and it is a three-step process: sorption into waxes, diffusion through the cuticular membrane (CM), and desorption into the apoplast and symplast of the epidermal cells. The first step (sorption) is considered to be the driving force for penetration^12^,^13^,^14^. Sorption and diffusion are difficult to differentiate because one is always accompanied by the other, and they are commonly combined to characterize penetration processes. It is well known that two parallel pathways exist in the cuticle: aqueous pores and the lipophilic pathway. Organic pollutants are mainly hydrophobic chemicals that diffuse through the cuticular lipids composing lipophilic pathway^8^,^15^,^16^,^17^,^18^. Studies have demonstrated that enhanced penetration can be achieved after dewaxing^12^,^19^. Therefore, Buchholz *et al*.^8^ divided the cuticle into two functional layers: a limiting skin (LS) comprising EW and part of the CP and a sorption compartment (SC) comprising the remaining CP and polysaccharides. Wild *et al*.^20^ found that PHE can be transported through spinach and corn leaf cuticles in 24–48 h, which is a relatively short period of time. Thus, the barrier function of the cuticles seems to be very limited. Riederer and his colleagues^21^ proposed a two-compartment sorption model in which the cuticle was divided into wax and cuticular matrix and was believed to have no significant effect on the sorption capacities after dewaxing. The fundamental principles of the two models were compared, and an obvious contradiction emerged in terms of the contributions of the cuticle components to accumulation. The ambiguity in the limiting effects of waxes and the remaining cuticle components indicates the necessity of further investigation.

The penetration mechanism relates to the plant uptake model for hydrophobic organic contaminants (HOCs). In partition-limited model for HOC plant uptake, Chiou *et al*.^22^ concluded with the plant lipids as the major reservoir for hydrophobic contaminants. After its establishment, many researchers have focused on the prediction of the organic contamination in the plant^23^,^24^,^25^,^26^. Due to the lack of the accurate measurement of plant lipid content, researchs have reported that the predicted contamination was lower that the measured one^27^,^28^. Then several efforts were involved to improve the existing models. For example, Zhu *et al*.^25^ included dechlorophylization into the lipids determination and replaced octanol-water partition coefficient with triolein-water partition coefficient, resulting in substantial improvements in the application of partition-limited models. Through the supplement of the carbohydrate contribution, Zhang *et al*.^28^ proposed a modified sorption prediction model and desired accuracy was gained. Recently, organic pollutants have been observed to exhibit continual cluster formation and growth in lipid media over time, resulting in localized “hot spots” in a living system^29^. This behavior is quite different from that described by the fundamental hypothesis of the conventional plant uptake model^22^,^23^,^24^,^25^,^26^,^27^,^28^. Chen *et al*.^30^ studied the sorption of polycyclic aromatic hydrocarbons (PAHs) by natural organic matter and observed a “melting away” effect exerted by concentrated small PAH molecules on the solid-amorphous waxes. According to their results, a melting transition occurred during the sorption of organic chemicals onto the cuticular waxes. A subsequent study investigated the distinct roles of several fractions of cuticles in PHE sorption^31^. Cutin was identified as the genuine dominant sorbent, not extractable lipids. Recently, a direct method was applied to verify the superior capacity of polymeric lipids (cutin) in trapping organic chemicals, and the affinity of the cuticle components to the organic pollutants was found to be the dominant driving force for the uptake. The liquid-like state of polymeric lipids was identified as a major contributor to pollutant trapping^18^. The chemical properties, cuticle composition and interactions at the interface between target chemicals and plant cuticles are factors that cannot be neglected when characterizing penetration. Moreover, penetration can easily be altered in this complicated and changing environment because the constituent ratios and structures can vary as the temperature and moisture change and be affected by interactions with coexisting compounds. Therefore, it is necessary to revisit the driving force and penetration mechanisms of organic chemicals in plant cuticles.

In most previous studies, a steady-state penetration system was applied, and Fick’s law was utilized to describe the penetration behavior of organic pollutants in plant cuticles. However, in a real environment, the difference in the organic pollutant concentrations between the donor and receiver decreases over time. Therefore, it would be inappropriate to discuss the driving force of the penetration and the contributions of cuticle components to the limiting effects in the steady state. The role of lipid components in the “partition-limited model” for the plant uptake of organic chemicals was underestimated, suggesting that more studies are needed to re-recognize the contribution of cuticular components in the accumulation and penetration of chemicals into plant surfaces. Furthermore, no technique is available for the direct measurement of the penetration rate in this heterogeneous membrane. The main objective of the current study is to probe the unsteady-state penetration behavior of organic pollutants in plant CMs *in vitro*, explore the componential contributions of the CM and re-analyze the existing penetration models. Finite dose diffusion characterization was applied, and sorption equilibrium-based calculations were conducted to quantitatively describe the penetration of a representative PAH, PHE, into the cuticle compartments.

## Results and Discussion

### Characterization of the green pepper CMs

**Figure 1:**
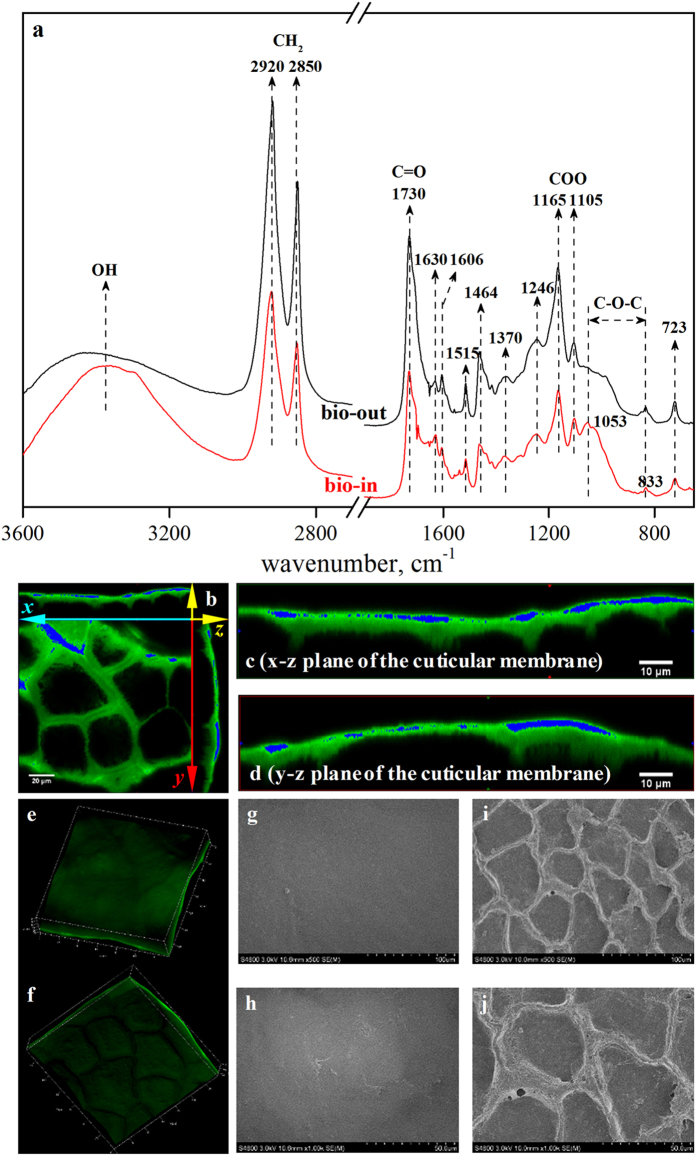
Selected ATR-FTIR spectra of the biological outer and inner sides (referred as bio-out and bio-in) of green pepper fruit CM (**a**), LCSM 3D micrographs of auramine O-stained CM (**e,f**) and their x–y, x–z, and y–z planes (**b–d**) and SEM micrographs of the outer (**g,i**) and inner surfaces (**h,j**) of the CM at different magnifications: 200× (**g,h**) and 500× (**i,j**).

The selected ATR-FTIR spectra of the biological outer and inner surfaces of the green pepper, a representative fruit, are presented in Fig. 1a. The CH_2_ units of the aliphatic components in waxes, cutin and cutan are shown by bands at approximately 2920, 2850, 1464, 1370 and 723 cm^−1 ^18,32,33,34. C=O stretching vibrations of ester bonds of cutin are ascribed to the peaks at approximately 1730 cm^−1^ with a shoulder at 1700 cm^−1^, which indicates the formation of hydrogen bonds. Aliphatic C–O–C, which corresponds to the oxygenated functional groups of polysaccharides, appear at approximately 1053 cm^−1^. Aromatic C=C and C=O assigned to the cutan fraction are also clearly indicated by the bands at 1606 cm^−1^. The relative distributions of lipid components and polysaccharides were further investigated through semi-quantification, as described in an earlier study^18^. The relative vibrational intensities of the aliphatic functional groups and polysaccharides were calculated. The ratios of the outer sides of the cuticles are higher than those of the inner sides (see Supplementary Table S1). Taking *ν*_*as*_ (−CH_2_)/*ν* (polysaccharides) for example, the ratio for the outer surface (4.30) is much higher than that for the inner surface (2.55). This suggested that the outer surface of the cuticle is generally aliphatic, whereas the inner surface of the cuticle is mainly covered by polysaccharides.

Topography of the CM was examined through scanning electron microscope (SEM). The outer surface of the CM was relatively smooth, and no obvious 3D wax micro-structure was observed (Fig. 1g,i). On the inner surface, irregular polygonal cuticular anticlinal pegs that had once covered the epidermal cells tightly *in vivo* were present (Fig. 1h,j). A 3D matrix of the CM was reconstructed through confocal laser scanning microscope (CLSM) (Fig. 1b–f). An obvious contrast in fluorescence intensities was evident: the middle layer of the cuticle architecture fluoresced strongly, whereas the side regions were relatively dim. To amplify this difference, the color “green_sat” was applied, resulting in sharply contrasting fluorescence intensities and a sandwich-like vertical distribution (Fig. 1b–d). Similar results were observed in another study investigating leaf cuticles^18^. The vertical stratification of three components (EW, CP and CL) in the plant cuticle was thus demonstrated, in agreement with the results of previous studies^7^,^10^,^18^. In combination with the FTIR results found here, this sandwich-like vertical distribution can be attributed to the differing lipophilic affinities of the cuticle components.

### Unsteady-state Penetration Characteristics

**Figure 2:**
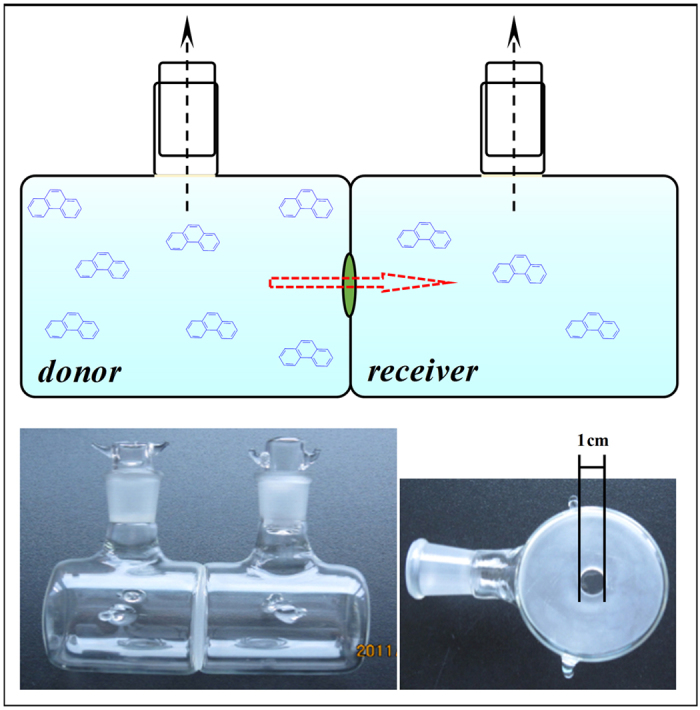
Penetration apparatus applied to plant cuticular membrane.

**Figure 3:**
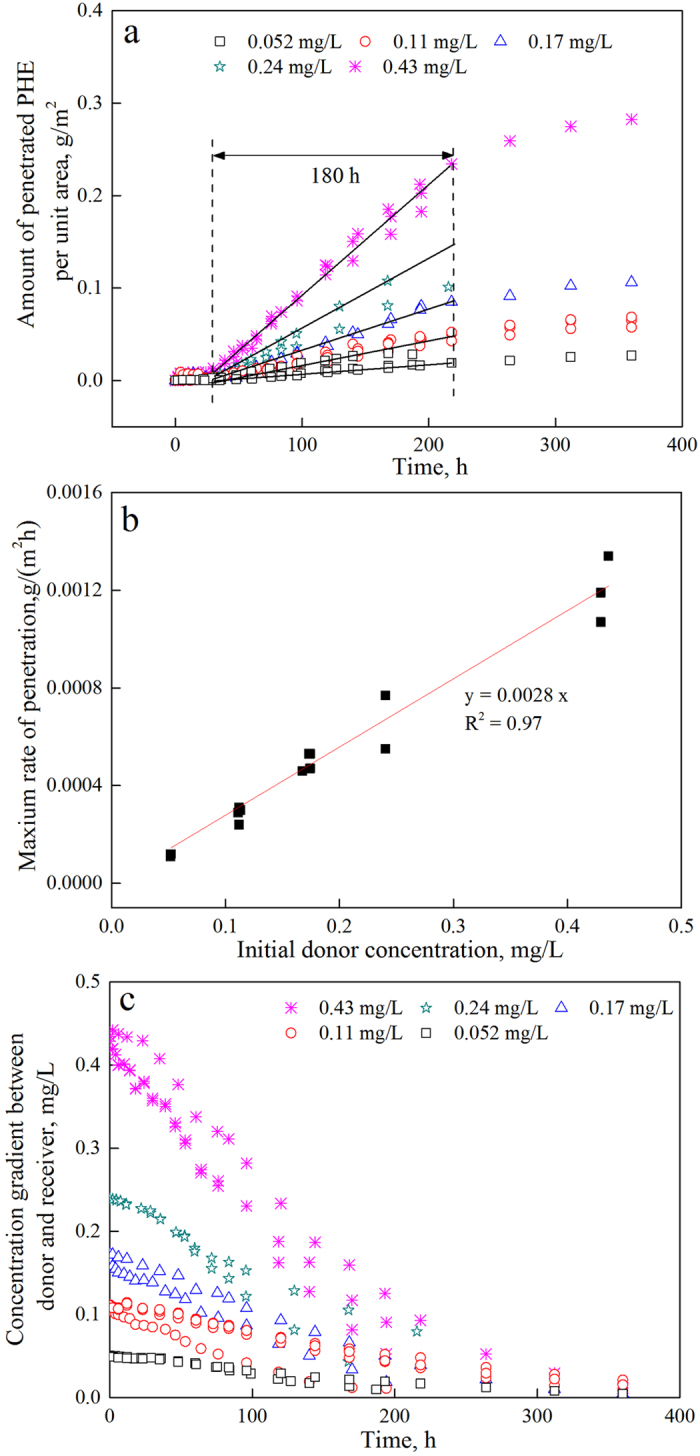
PHE penetration through green pepper fruit CM with different initial donor concentrations. (**a**) Time-courses of PHE penetration, (**b**) effect of initial donor concentrations on the maximum PHE-penetration rate, and (**c**) concentration gradient time-courses between the donor and receiver.

A customized apparatus was utilized in all the penetration experiments carried out in the current study. The structure diagram and photograph of the apparatus were presented in Fig. 2. In the current study, green pepper fruit CM was chosen as a model cuticle matrix, and PHE was selected as a representative pollutant molecule. The unsteady-state penetration kinetics of PHE with different initial donor concentrations were investigated (Fig. 3). Penetration time-courses were analyzed on an individual CM basis. The amount of penetrated PHE per unit area (*M*_t_/*A*, g/m^2^) and relative penetration ratio (*M*_t_/*M*_0_, %) can be calculated with the following equations:


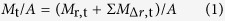



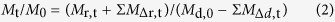


where *M*_d,t_ and *M*_r,t_ are the amounts of PHE in the donor and receiver cell at time *t*, respectively, Σ*M*_Δd,t_ and Σ*M*_Δr,t_ are the sampling losses at time *t*; and *A* is the area of the CM (7.85 × 10^−2^ m^2^).

The sigmoidal penetration time-courses were characterized by three phases, and they fitted well in the finite dose diffusion system (Fig. 3a). Specifically, the penetration rate increased in the initial phase (lag phase), remained constant after being maximized in the second phase (maximum penetration rate phase) and decreased in the third phase (penetration plateau phase) when the penetration tended to reach equilibrium. Interestingly, the penetration in the second phase is nearly linear and exhibited a relatively long period compared with the other two phases. The penetration rates (*J,* g·m^−2^·h^−1^) of PAHs in plant cuticles and the penetration coefficients (*P,* m·s^−1^) can be calculated using the following equation:


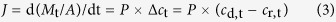


where *M*_t_ represents the amount of penetrated PHE (mg), and Δ*c* represents the concentration gradient between the cuticles (Δ*c* = *c*_d_−*c*_r_, mg/L). The slope of the linear penetration regression equation represents the maximal penetration rate, and the intercept of the x-axis reflects the hold-up time, which is the time period before pollutant diffuses into the receiver cell (see Supplementary Table S1).

It is commonly acknowledged that the maximal penetration rate and time lag are two essential factors that define the penetration performance. PHE was held up in the cuticle matrix for approximately 18–48 h (Fig. 3a), until it was detected in the receiver cell. This period was similar to those in the *in vivo* system (24–48 h)^20^, indicating that organic pollutants can penetrate through the cuticle rapidly and enter epidermal cells. The differences in penetration rates between the selected initial donor concentrations were quite significant (Fig. 3a), and a linear increase of *J*_max_ was observed as the concentration of the initial donor PHE solution increased (Fig. 3b). The corresponding regression equation of this linear increase is *J* = 0.0028 × *C*_d,0_ (R^2^ = 0.97, *n* = 14). The penetration coefficient (*P*) is 7.7 × 10^−7^ m · s^−1^, which is much higher than that of the 2,4-D penetrations in the green pepper fruit cuticle (~10^−8^ m · s^−1^)^35^. Notably, the linear penetration times for each initial donor concentration were nearly the same (approximately 180 h, see Fig. 3a), and the relative penetration ratios (*M*_t_/*M*_0_) also seem to be independent of the initial donor concentrations (see Supplementary Fig. S1).

According to Fick’s Law, the concentration gradient (Δ*C*) is the dominant driving force for penetration in a steady-state system^8^,^36^. Knoche *et al*.^37^ investigated the finite dose diffusion of 2-(1-naphthyl) acetic acid (NAA) in isolated tomato cuticle and ascribed the linear penetration of NAA to the constant concentration gradient, which was identified as the stable driving force of penetration. The concentration gradient was stabilized by maintaining the donor solution in a saturated state, and the NAA concentration in the receiver was ignored. However, herein, the concentration gradient between the donor and receiver gradually decreased as penetration progressed, and when the linear penetration phase ended, the concentration gradients decreased 3- to 6-fold relative to the initial ones (Fig. 3c). Thus, no constant concentration gradient is available to serve as a driving force, and it is difficult to assign the linear penetration phenomenon to the concentration gradient between the donor and receiver.

**Figure 4:**
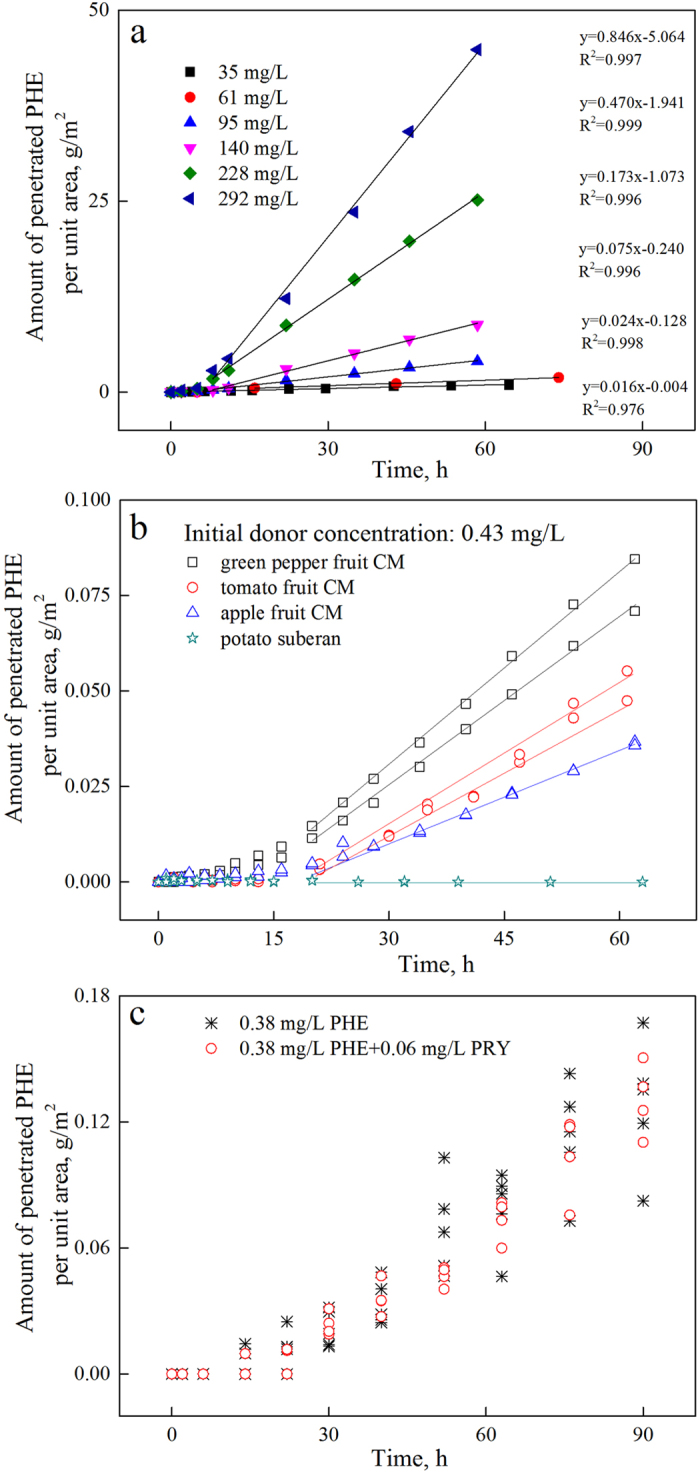
Time-course of TCP penetration through green pepper fruit CM (**a**); PHE penetration through green pepper, tomato and apple fruit CMs and potato periderm (**b**); and PHE penetration through green pepper CM in the absence and presence of 0.06 mg/L PYR as a competitor (**c**).

To explore the generality of this unusual phenomenon, another hydrophobic organic pollutant, 2,4,6-trichlorophenol (TCP), was chosen, and its unsteady-state penetration characteristics were investigated (Fig. 4a). Linear penetration was also observed with the selected initial concentrations, and the penetration rate increased as the concentration of the initial donor pollutants increased. Subsequently, three CMs and a periderm from different plants were isolated and employed in unsteady-state penetration experiments of PHE (Fig. 4b). These CMs and periderm were selected from common fruits and vegetables, and their contamination could lead to severe health risks and hidden toxicity. As presented in Fig. 4b, linear penetration of PHE occurred in all three CMs, and the penetration rates differed. Throughout the observation period, no pollutant was detected in the receiver cell of the potato periderm, possibly because of its large thickness and dense structure. The penetration rates exhibited the following order: green pepper CM (0.0016 g·m^−2^ · h^−1^) > tomato CM (0.0012 g·m^−2^·h^−1^) > apple CM (0.00079 g·m^−2^ · h^−1^). In a previous study^31^, the sorption capability of apple cuticle (*K*_d_, 54,017 L/kg) was found to be higher than that of green pepper (48,880 L/kg) and tomato (40,747 L/kg). Thus, higher sorption capacities do not correlate with stronger hindering or buffering effects. In contrast, as the sorption capacity increases, the linear penetration time also increases, while the penetration rate decreases (the linear penetration time of apple is nearly twice that of green pepper, see Supplementary Fig. S2).

The transport behavior of organic pollutants was found to be much more complicated in the real environment. For instance, various pollutants coexisted, and when they came together at the same bio-interface, competitive adsorption may occur, potentially reducing the contamination risk of living organisms. To explore this complexity, the competitive penetration of two solutes (PHE and PYR) was initially investigated (Fig. 4c). No obvious difference was observed after the addition of PYR, indicating that no competitive effect exists between these two compounds. PAHs penetrate into the cuticle through a dissolution and diffusion process, and as observed in the single-solute penetrations, linearity occurred periodically during the penetration time-course. In a study investigating the single-solute and bi-solute sorption of PHE and PYR onto raw and modified pine bark^38^, similar sorption isotherms of PHE and PHE with and without PYR were obtained, which implied no obvious competitive effect between these two PAHs with regard to sorption.

### Sequential Penetration within the Three Compartments

In the absence of a stable external driving force, the root cause of linear penetration is unclear. The CM has been stratified into three adjacent compartments, and several studies have demonstrated their differing affinities to organic pollutants^14^,^18^,^32^,^39^. Given the difficulty of quantifying the PHE absorbed in each component directly, PHE was analyzed through mass balance. Previous research has shown that the adsorption and desorption at the cuticle interface is fast and that the rate-limiting step is the diffusion in the cuticle^12^,^40^. Therefore, PHE at the interface of the donor solution (*c*_d,t_) and the EW (*Q*_EW,t_) was assumed to reach adsorption equilibrium, and a similar assumption was made for the PHE at the interface between CL (*Q*_CL,t_) and the receiver solution (*c*_r,t_). Additionally, several approximations were required because of the lack of *K*_d_ values for the cuticle components. *K*_d,PC1_ (48,880 L/Kg) was determined and employed to represent *K*_d,EW_ because its sorption capability was equivalent to those of intact components after dewaxing^34^,^41^,^48^,^49^,^52^. This value was calculated through the regression of the sorption isotherm of intact green pepper fruit cuticle. *K*_d,PC3_ (7090 L/Kg) was substituted for *K*_d,CL_^32^.

**Figure 5:**
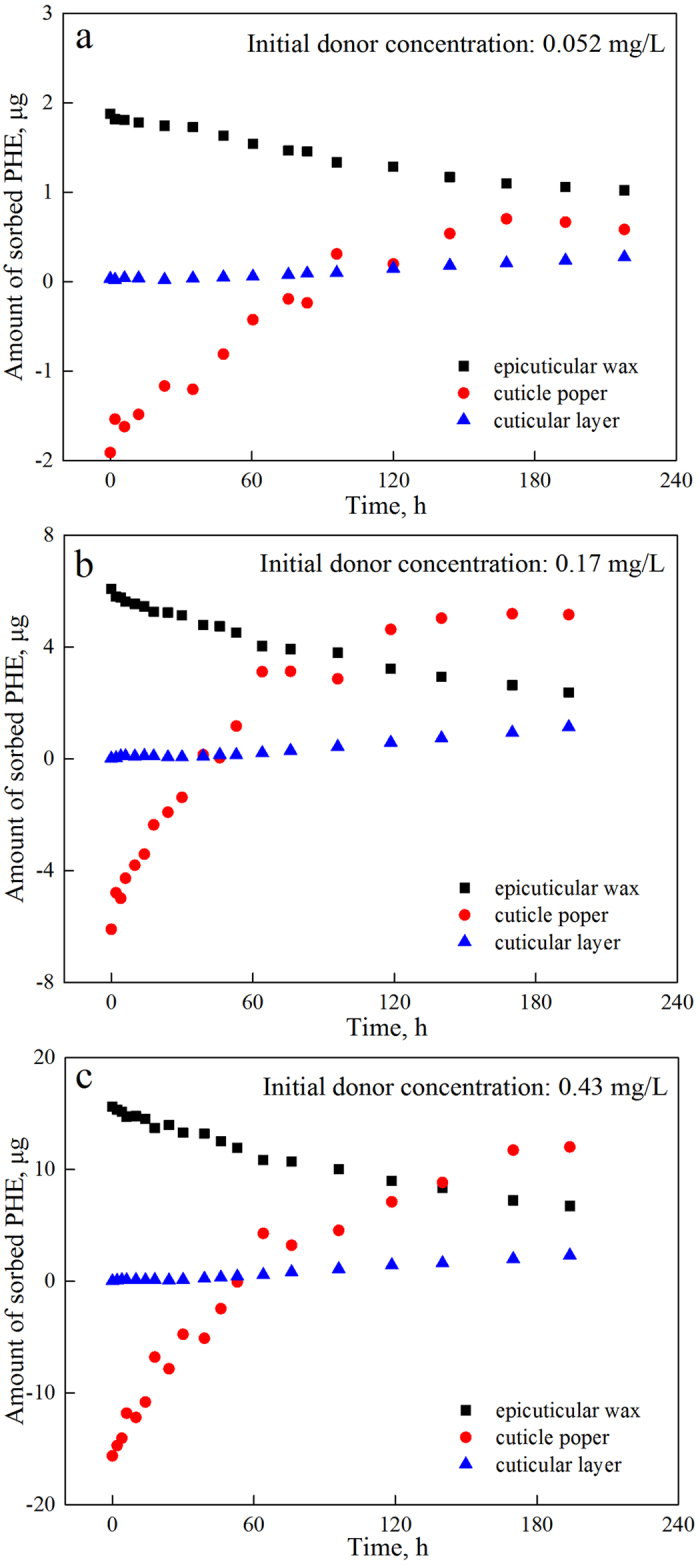
Amounts of PHE sorbed in epicuticular waxes (EW), cuticle proper (CP) and cuticular layer (CL) of green pepper fruit cuticular membrane (CM) as a function of time with selected initial donor concentrations.

The accumulated amounts of PHE in the CM (*M*_cuticle_), EW, CL and CP can be calculated by the following equations:


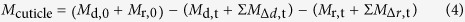



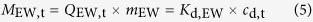



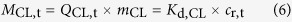



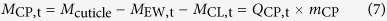


where *m*_cuticle_ is the average weight of the applied cuticles (12 ± 1.3 mg). According to the composition of the CM, the weights of EW (6.2%), CP (composed of cutin, 64.8%) and CL (composed of cutan and polysaccharides, 29.0%) were calculated to be approximately 0.744, 7.776 and 3.48 mg. The time-courses of PHE penetration into the three compartments with selected initial concentrations are presented in terms of amounts in Fig. 5(a–c) and densities (see Supplementary Fig. S3 a–c). Throughout the penetration, the amount of PHE in EW was first reduced and then maintained, whereas those in CP and CL increased until achieving equilibrium. The accumulated amounts of PHE in CP were negative for all selected initial concentrations before 60 h because the calculations were conducted based on sorption equilibrium at the interface between the donor-EW and receiver-CL, although equilibrium had not yet been achieved. Notably, PHE preferred to accumulate in CP rather than in CL, as shown by both the rate (slope of the curve) and the amount. The amounts of PHE in CP exceeded those in EW before 218 h, when the linear penetration was complete, and this difference is attributed to the larger proportion of CP than EW. The PHE densities in EW were higher than those in CP and CL throughout penetration.

**Figure 6:**
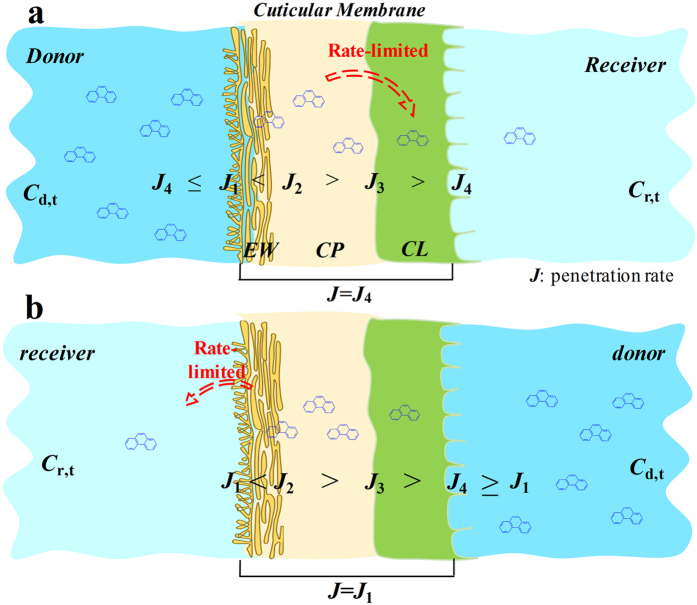
Sequentially forward (**a**) and reverse (**b**) penetration of phenanthrene in three compartments of cuticular membrane.

Because of the uneven vertical distribution of the cuticle components, the diffusion rates of pollutants in the CM fluctuated. Therefore, the penetration rate through the CM (*J*) can be periodically analyzed. The penetration rate from the donor to EW was *J*_1_, EW to CP was *J*_2_, CP to CL was *J*_3_ and CL to receiver was *J*_4_. We created a schematic diagram of the sequential penetration of PHE into the three compartments of the CM (Fig. 6a). The penetration first increased and then plateaued, indicating that *J*_1_ ≥ *J*_4_ and *J* = *J*_4_. As the penetration proceeded, PHE in EW decreased and accumulated in the other two compartments; thus, *J*_2_ > *J*_1_ and *J*_2_ > *J*_3_ > *J*_4_. During the linear penetration, the penetration rate remained steady at its maximum value (*J*_4_ remained unchanged). The penetration from CL to the receiver is a desorption equilibrium process. Abundant studies have confirmed that EW and CP possess much stronger sorption capacities for organic pollutants than CL^31^,^32^,^38^,^39^,^41^. In this dynamic sorption and desorption process, the rate-controlling step is the translocation of PHE from CP to CL (*J*_3_), which slowed down the whole process.

**Figure 7:**
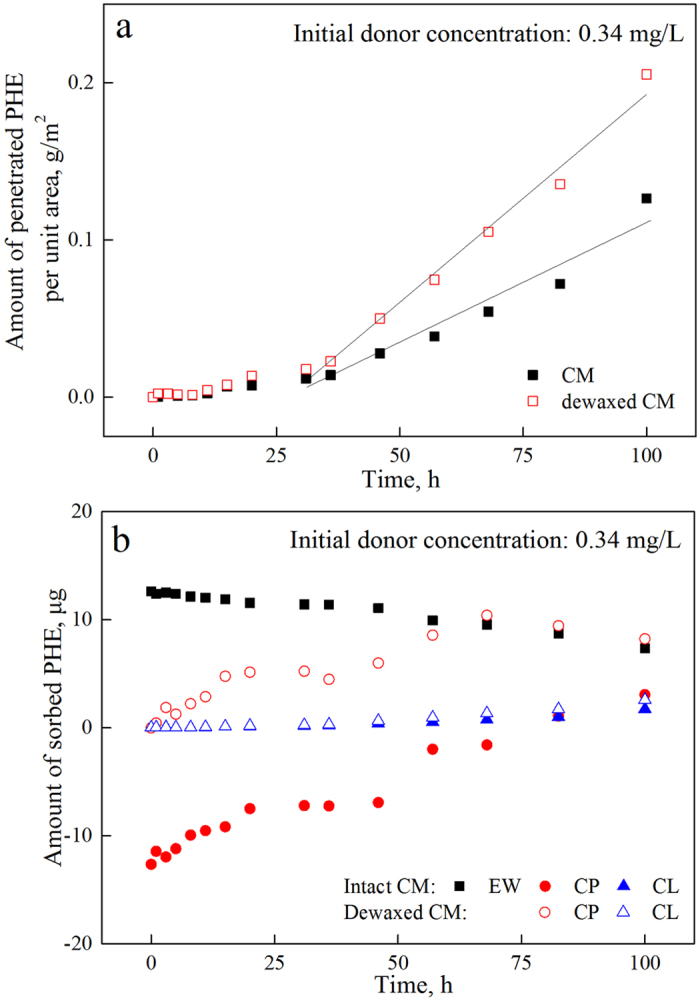
PHE penetration through green pepper fruit CM with a selected initial donor concentration (0.34 mg/L) as a function of time: (**a**) Total PHE penetration and (**b**) PHE amounts in EW, CP and CL.

Previous studies have asserted the significance of EW in the penetration of organic pollutants into plants because of its barrier effect based on the considerable enhancement of the penetration rate after dewaxing^42^,^43^,^44^. We simulated this process by dewaxing the green pepper fruit CM and compared its PHE penetration with that of the intact fruit CM (Fig. 7). With an initial donor concentration of 0.34 mg/L, the dewaxed CM showed more PHE accumulation than intact fruit with nearly twice the amount of accumulated PHE (Fig. 7a), which is consistent with previously mentioned results. Time-courses of penetration in various compartments are presented in Fig. 7b.

After dewaxing, the accumulation of PHE in CP was efficiently enhanced, whereas that in CL was not significantly different. Enhancement of the penetration was seen only in CP and did not extend to CL. The higher sorption capability of CP resulted in its superior hindering effect, which was greater than that of EW. From this point of view, although the role of EW as an efficient contamination barrier was not impaired, it should not be assumed that EW can protect the whole organism alone. A study investigating the bilateral desorption of 2,4-D from CM revealed obvious asymmetry: most of the 2,4-D in the CM desorbed from the inner surface, even after dewaxing, whereas only a small quantity desorbed from the outer surface and exhibited a much slower desorption rate^45^. The chemicals captured by the middle-embedded polymeric lipids had much more difficulty escaping from one side than those in the EW barrier. The superior organic pollutant-sorption capacities of EW and CP slowed the diffusion of organic pollutants but created sharp concentration gradients within the plant cuticular membrane, which likely supplied a stable driving force and eventually caused linear penetration. This endogenous driving force increased as the penetration rate of pollutants into CP, which mainly consisted cutin, a superior reservoir for organic pollutants, increased^49^,^52^.

Organic pollutants in the environment are well known to be dynamically transported among various environmental media, and this dynamic transport is temperature sensitive and could alter local pollution levels^46^,^47^,^48^. For example, during daytime in the summer, the organic pollutant concentrations of plants become diluted as the temperature decreases; conversely, during nighttime in the winter, the organic pollutant concentrations of plants increase as the temperature increases^49^,^50^. Similar phenomena are known, including the latitude effect^51^ and grasshopper effect^52^. The exhalation of organic pollutants from plants is feasible, as it is their inhalation into plants. Therefore, there are at least two aspects that warrant further research.

**Figure 8:**
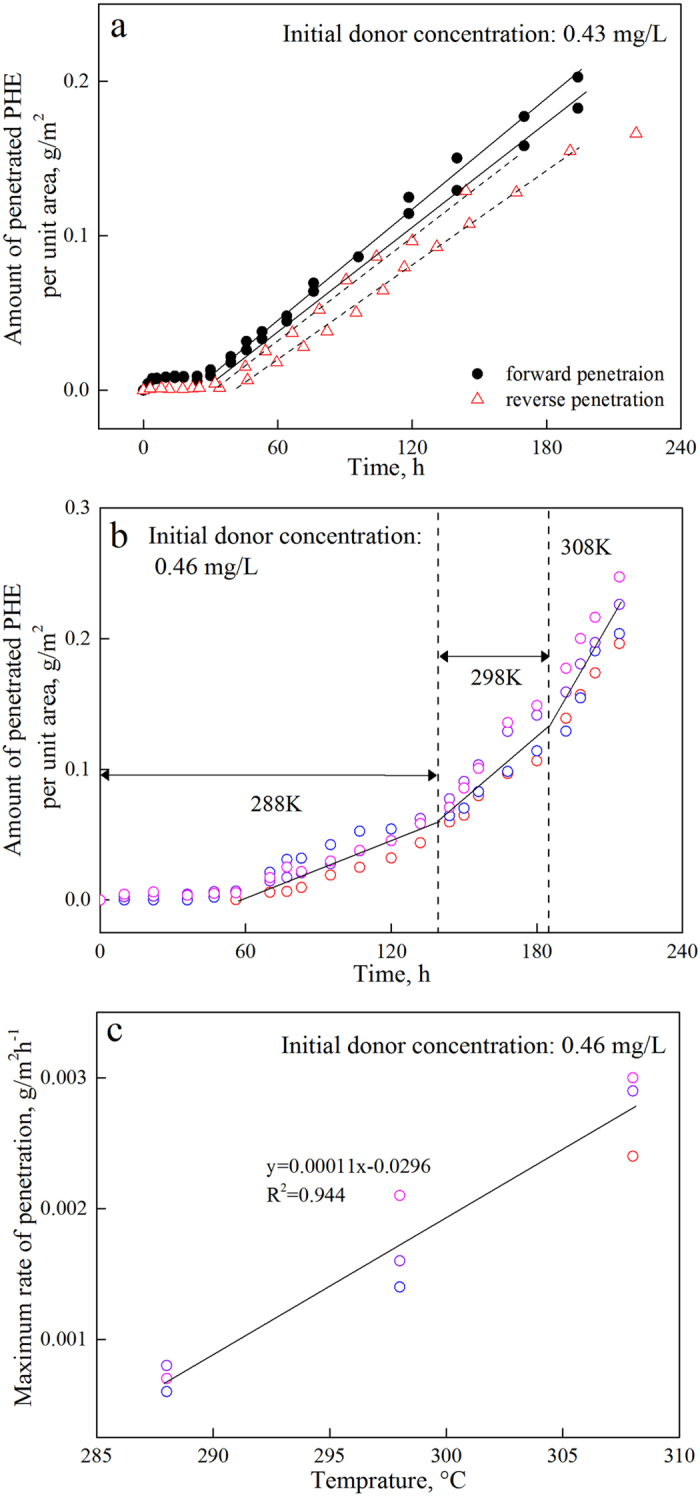
Time-courses of PHE forward and reverse penetration through green pepper fruit CM (**a**), time-courses of PHE through green pepper fruit CM under different temperatures (**b**), and effect of temperature on the maximum penetration rate (**c**).

First, to simulate the exhalation of organic pollutants from the plant to the ambient environment, the reverse penetration of PHE from the biological inner surface to the outer surface was investigated. As presented in Fig. 8a, the reverse penetration curves were almost the same as those of the forward process, and the penetration rate and hold-up time were similar. The transport of organic pollutants across the cuticular interface is therefore confirmed to be a reversible process. The reverse penetration is schematically illustrated in Fig. 7b and is similar to the forward process. In this process, *J*_4_ > *J*_1_ and *J* = *J*_1_ as the PHE accumulation in CP increases. The PHE in CL decreased, indicating that *J*_3_ > *J*_2_ > *J*_1_. As the penetration proceeded, the PHE accumulated in CP and EW, and as shown by the density, after 120 h, the PHE concentration in EW exceeded that in CL (see Supplementary Fig. S4). This “active transport” cannot occur spontaneously and can be ascribed to the sharp concentration gradient created between the cuticular components. The amount of PHE in EW during reverse penetration is far smaller than that of the forward process with the same initial donor concentration because of CP’s superior accumulating ability.

The impact of temperature on the penetration process was simulated using a temperature-programmed system, and three environmentally relevant temperatures were investigated (Fig. 8b,c). With an initial PHE concentration of 0.46 mg/L, the slopes of the penetration curves became sharper as the temperature increased; during each period of a given temperature, the accumulation was linearly fitted. The regression functions of the six parallel penetrations are listed and can be found as see Supplementary Table S2, which shows that the penetration rate increased 3.0- to 4.4-fold from 15 °C to 35 °C. Recently, we investigated the glass-phase transition of a leaf cuticle and found that at temperatures of 15 °C to 35 °C^18^, waxes were in an amorphous solid state, and polymerized lipids (mainly cutin) were in a liquid-like viscous state. Elevating the temperature accelerated the Brownian motion and substantially promoted penetration. This reasoning is in good agreement with the related free volume theory as the temperature increases^8^. The size of the temporary holes in the cuticular matrix increased, and solute hopped into them during the rearrangement of the free volume. In addition to the enlargement of the free volume, the activation energy required for organic pollutant hopping was suppressed, and the penetration rate was thus elevated. The penetration rate increased as a function of temperature, and the variability among the individual CMs was enlarged. This may have been caused by the increasingly variable composition of the cuticle and the elevation of the randomness as the temperature increased. The facilitated liquidity of polymeric lipids and enlarged side of the temporary holes in the CM during the temperature elevation could both contribute to the transport of the chemicals.

As mentioned previously, chemical computational methods have indicated that after introduction into lipid media, the uniformly distributed PAHs exhibit continual cluster formation and growth over time^29^. When this clustering occurs in a living system, it could result in localized “hot spots”^20^,^29^. Highly concentrated organic pollutants in plant lipids could lead to local melting of the lipophilic matrix^30^. The resulting phase transition could then promote pollutant penetration because of the fast molecular motion in the quasi-liquid state. It could be inferred that intramolecular interactions of organic pollutants contribute to the formation of lipophilic pathways for their own penetration and diffusion. In the current study, the high concentration resulted from retention in polymeric lipids, and it is possible that the stable penetration resulted from the intramolecular interactions of pollutants themselves and was enhanced by the accumulative nature of the polymeric lipids in the cuticle. The liquid-like polymeric lipids (cutin) assembled to form siphonic pipes (i.e., lipophilic pathway) for HOC penetration through the cuticular membrane of plant. The temperature elevation could intensify this siphon process and make this lipophilic pathway more effective and rapid.

In summary, the unsteady-state penetration of PHE in a finite dose system was investigated by dividing the CM into three compartments, and a modified penetration model was illustrated based on this compartmentalization. The preferential barrier effect of CP is highlighted as a superior accumulation reservoir because of its strong sorption capability in a quasi-liquid state. The high sorption capability and accelerated penetration rate result in the efficient retention of organic pollutants. The general suitability of the proposed model was verified with another hydrophobic pollutant and several CMs. Determining the endogenous driving force for the linear penetration phenomenon could extend our insights regarding the uptake and transfer behavior of semi-volatile organics at the surfaces of plants. Moreover, the proposed modified model offers more accuracy in the prediction of pollutant sorption and partitioning at air/water–plant interfaces.

## Materials and Methods

### CM Isolation and Characterization

Cuticular membranes (CMs) were isolated enzymatically according to the procedure described by Orgell^53^ and modified by Yamada *et al*.^54^ Briefly, epidermal discs (15 mm in diam.) were excised, selected to be free of visual defects, and then incubated in a mixture of cellulose (w/v, 2%), pectinase (w/v, 2%), and NaN_3_ (1 mM) in citric acid buffer (20 mM, pH 3.8) at room temperature. The enzyme solutions were refreshed periodically until the CMs fully separated from the tissue. Subsequently, the CMs were washed extensively with deionized water, air-dried and stored at room temperature until use. Dewaxed CMs were acquired by rinsing the CM surface with dichloromethane (CH_2_Cl_2_) for 1 min. The residual organic solvent was removed by air suction.

A Nicolet 6700 FTIR spectrometer (Thermo Scientific, USA) equipped with an attenuated total reflectance (ATR) accessory (iS10 Smart iTR) was utilized to record the ATR–FTIR spectra of the morphological outer and inner surfaces of the CMs in the range of 4000–650 cm^−1^. The ATR accessory allows the direct examination the intact structures of samples in the solid or liquid state without further preparation. The resolution was set at 1.0 cm^−1^. The surface morphology of a green pepper was examined using a field emission scanning electron microscope (FE-SEM, S-4800; Hitachi, Tokyo, Japan) with an accelerating voltage of 3 kV. Specimen preparation for scanning electron microscopy (SEM) imaging was performed as described in an earlier study^18^. Briefly, air-dried CMs were directly frozen with liquid nitrogen (−196 °C) and then vacuum desiccated. Before scanning, a thin layer of platinum was sputter coated. The 3-dimensional (3D) structure of the CM of a green pepper fruit was reconstructed by imaging an auramine O (Sigma; 0.01% w/v in 0.05-M Tris/HCl, pH 7.2)-stained CM under a confocal laser scanning microscope (CLSM). Auramine O is a lipophilic fluorescent dye that has distinct affinities to cuticle components and is commonly used to differentiate lipophilic components^55^,^56^. Cuticles were stained for 15 min and rinsed thoroughly with distilled water. Slides were mounted with the outer side upward in distilled water with a cover slip and viewed quickly (the sample standby time was less than 1 h). A Zeiss LSM 710 NLO CLSM with a Plan-Apochromat 63×/1.4 DIC oil-immersion objective (Zeiss) was used for auramine O imagine with excitation at 430 nm and detection at 500 nm. 3D images of the reconstructed cuticle were acquired by z-stack projections with a vertical resolution of 0.35 μm.

### Penetration Experiments

Finite dose diffusion experiments were performed using a modified version of an earlier method described by Knoche and Bukovac^57^. In detail, CMs were mounted between two glass diffusion half-cells, and the contacted sides consisted of ground glass with a round hole (1 mm diam.). The cells were fixed with enough pressure to prevent solution leakage along the contact sides (Fig. 2). The morphological outer side of the CM faced the donor cell, and the inner side faced the receiver cell. All CMs and diffusion cells were checked for leaks according to the following procedure. The receiver cell was filled with background solution, while the donor cell was kept empty. The background solution comprised 0.01 mol/L CaCl_2_ (to maintain a constant ionic strength) and 200 mg/L NaN_3_ (as a biocide). Only cells and CMs on which no visual water diffusion was observed over a period of 3 h were used. PHE was dissolved in the background solution at different concentrations (0.05–0.43 mg/L) to generate donor solutions.

Penetration experiments were initiated by adding approximately 150 mL of PHE and background solution to the donor and receiver cells, respectively. The cells were filled to minimize the headspace volumes and avoid evaporation. Parallel apparatuses were agitated in the dark at 25 ± 0.5 °C to minimize boundary layers and avoid PHE photolysis during the penetration process. Pyrene (PYR) was chosen as a competitor for PHE to investigate the competition effect in penetration. The unsteady-state penetration characteristics of TCP, a representative hydrophobic organic compound, through green pepper CM and that of PHE through isolated tomato, apple and potato CMs were also investigated. The temperature effects were examined by gradually heating during the penetration process. Specifically, penetration before 144 h was maintained at 15 °C, the period between 144 and 192 h was maintained at 25 °C, and that between 192 and 227 h was maintained at 35 °C. In each period, 4 to 17 samples were taken. The characteristics of reverse penetration were also investigated for comparison with those of forward penetration. Solution samples (0.3–0.5 mL) were taken periodically from both the donor and receiver cells and then mixed with 0.5 mL of acetonitrile for high-performance liquid chromatography (HPLC) analysis. PHE concentrations were measured with an Agilent 1200 HPLC equipped with a G1321A fluorescence detector and Agilent Eclipse XDB-C 18 column (4.6 mm × 250 mm × 5 μm). Injection volumes of 15 μL, a mobile phase of 90% acetonitrile/10% water with a flowrate of 1 mL/min, an excitation wavelength of 244 nm and an emission wavelength of 360 nm were chosen for PHE elution separation and detection.

### Extraction of PAHs from CMs

After the penetration experiment, the CMs were rinsed with deionized water to remove unabsorbed PHE from their surfaces, and then, they were air-dried and weighed. PHE was extracted using a 1:1 mixture of n-hexane and acetone in a circulating water bath in an ultrasonicator for 30 min. The extract was collected, and new solvent was added each time. The procedure was repeated 3 times to ensure complete extraction. The obtained extract was concentrated at 40 °C using a vacuum rotary evaporator, transferred with n-hexane to a silica column (2.5 g silica filled), and then eluted with 25 mL of a 1:1 mixture of n-hexane and dichloromethane. The eluate was concentrated to near dryness at 40 °C by vacuum rotary evaporation and then transferred with 3 mL of acetonitrile and filtered through a syringe filter (0.22 μm) into a 2-mL sample bottle for HPLC analysis. Note that before each vacuum rotary evaporation, 30 μL of dimethyl sulfoxide was added to the solution to protect PHE from evaporating with the organic solvent.

## Additional Information

**How to cite this article**: Li, Y. *et al*. Organic Pollutant Penetration through Fruit Polyester Skin: A Modified Three-compartment Diffusion Model. *Sci. Rep.* **6**, 23554; doi: 10.1038/srep23554 (2016).

## Supplementary Material

Supplementary Information
