# Supplementary material for: Organic Pollutant Penetration through Fruit Polyester Skin: A Modified Three-compartment Diffusion Model
Source: Sci Rep. 2016 Mar 24;6:23554. doi: 10.1038/srep23554 (PMC4806373; doi:10.1038/srep23554)
Supplement: Supplementary Information [file srep23554-s1.pdf]

Supplementary Information for *Scientific Reports*

Organic Pollutant Penetration through Fruit Polyester Skin: A Modified Three-compartment Diffusion Model

Yungui Li, [†,‡] Qingqing Li, [†,§] and Baoliang Chen[*,†,§]

† Department of Environmental Science, Zhejiang University, Hangzhou 310058, China

‡ key Laboratory of Solid Waste Treatment and Resource Recycle, Ministry of Education, Southwest University of Science and Technology, Mianyang 621010, China

§ Zhejiang Provincial Key Laboratory of Organic Pollution Process and Control, Hangzhou 310058, China

* Corresponding Author E-mail: blchen@zju.edu.cn

Phone: 0086-571-88982587

Fax: 0086-571-88982587

Supplementary Information consists of 7 pages including this one.

There are three Tables and four Figures.

December 7, 2015

Table S1. Relative Contents of Selected Aliphatic Functional Groups/Polysaccharides in the Outer Side (o) and the Inner Side (i) of Cuticular Membranes of Green Pepper.

cuticle	ν_{as}(-CH_2)/ ν(polysaccharides)[a]	ν_{as}(-C=O)/ ν(polysaccharides)[a]	ν(-COO^-)/ ν(polysaccharides)[a]
bio-o	4.30	2.63	2.24
bio-i	2.55	1.60	1.38

[a] *The* intensity ratio of the band at 2920 cm^{-1} for –CH_2 to the band at 1053 cm^{-1} for polysaccharides, the intensity ratio of the band at 1730 cm^{-1} for –C=O to the band at 1053 cm^{-1} for polysaccharides, and the band at 1165 cm^{-1} for –COO^- to the band at 1053 cm^{-1} for polysaccharides were calculated and used to quantify the relative content of aliphatic components and polysaccharides.

Table S2 Linear Penetration Equations of Phenanthrene Across Green Pepper Fruit Cuticular Membrane and the Hold-up Time

Initial concentration, mg/L	Regression equation	R^2	R_{max}, g/（$m^2·h$）	Hold-up Time, h
0.43	y = 0.00107 x - 0.02149	0.996	0.00107	20
0.43	y = 0.00119 x - 0.02326	0.996	0.00119	20
0.44	y = 0.00134 x - 0.04060	0.997	0.00134	30
0.24	y = 0.00077 x - 0.02182	0.997	0.00077	28
0.24	y = 0.00055 x - 0.01414	0.996	0.00055	18
0.17	y = 0.00046 x - 0.01317	0.994	0.00046	29
0.17	y = 0.00047 x - 0.01853	0.991	0.00047	39
0.17	y = 0.00053 x - 0.01592	0.978	0.00053	30
0.17	y = 0.00053 x - 0.01035	0.981	0.00053	20
0.11	y = 0.00031 x - 0.00532	0.987	0.00031	17
0.11	y = 0.00030 x - 0.00963	0.998	0.0003	32
0.11	y = 0.00029 x - 0.00939	0.997	0.00029	32
0.11	y = 0.00024 x - 0.00830	0.994	0.00024	35
0.052	y = 0.00012 x - 0.00311	0.984	0.00012	26
0.052	y = 0.00011 x - 0.00395	0.989	0.00011	36

Table S3 Linear Penetration Equations of Phenanthrene Across Green Pepper Cuticular Membrane Under Different Temperatures

parallel samples	15 °C		25 °C		35 °C		ratio increase *
	linear regression equation	R^2	linear regression equation	R^2	linear regression equation	R^2	
1	y = 0.0007x - 0.0476	0.967	y = 0.0016x - 0.1759	0.966	y = 0.0024x - 0.3221	0.997	3.4
2	y = 0.0006x - 0.0133	0.953	y = 0.0014x - 0.1327	0.994	y = 0.0029x - 0.4098	0.844	4.8
3	y = 0.0008x - 0.0479	0.968	y = 0.0016x - 0.147	0.983	y = 0.0029x - 0.3839	1.000	3.6
4	y = 0.0007x - 0.033	0.974	y = 0.0021x - 0.2302	0.982	y = 0.003x - 0.387	0.999	4.3

*Initial concentration of phenanthrene was 0.46 mg/L; ratio increases were penetration rate under 35 °C to those under 15 °C ratios.

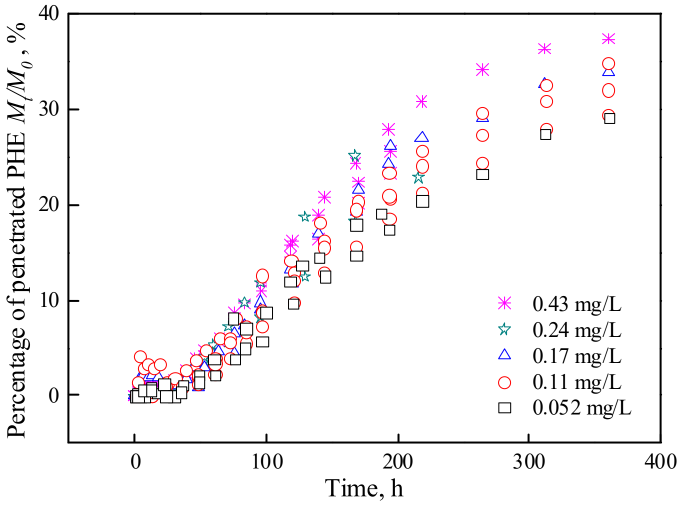

Figure S1. Relative penetration ratios of phenanthrene through green pepper fruit cuticular membrane as a function of time.

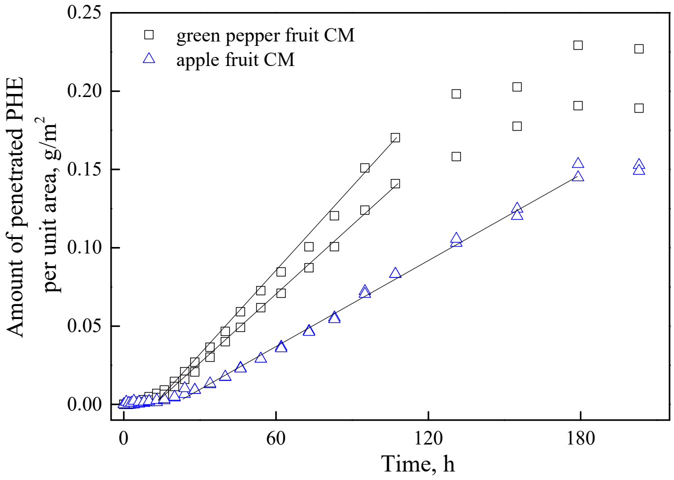

Figure S2. PHE penetration through green pepper and apple fruit CMs

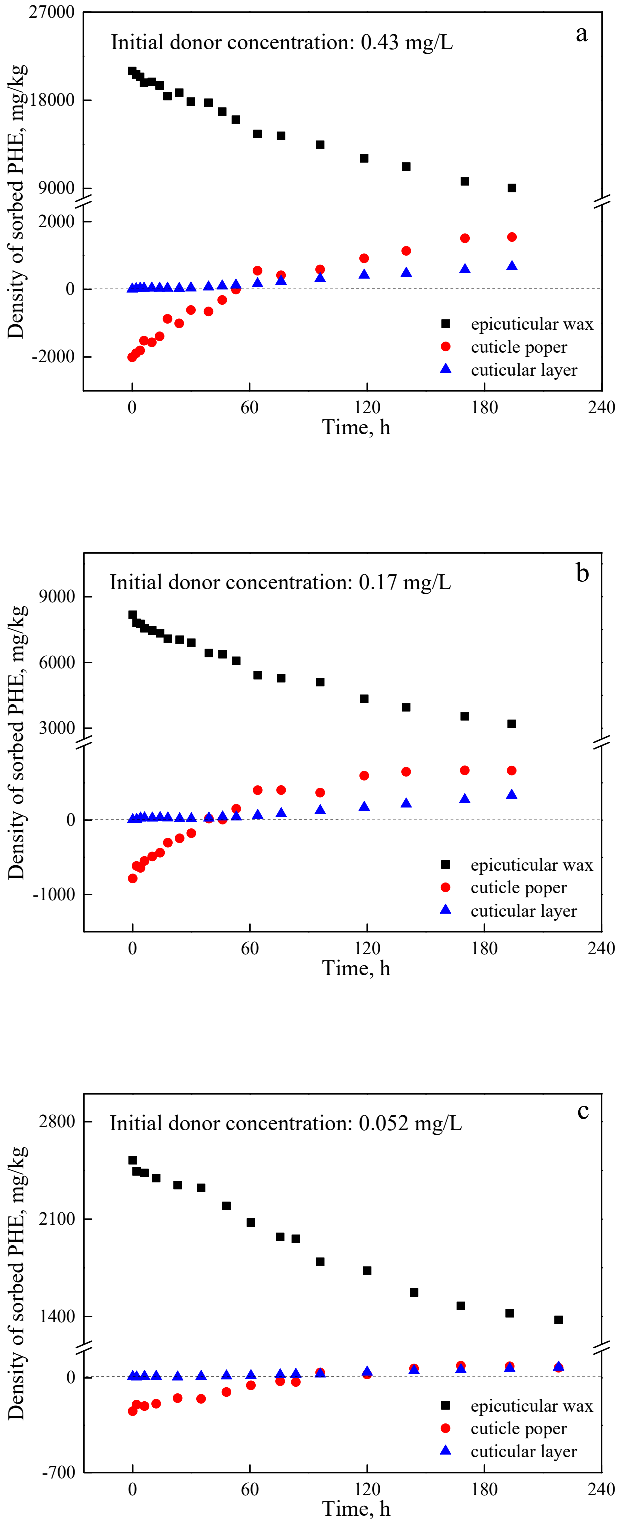

Figure S3. Density of phenanthrene absorbed on epicuticular waxes (a), cuticle proper (b) and cuticular layer (c) of green pepper fruit cuticular membrane during penetration

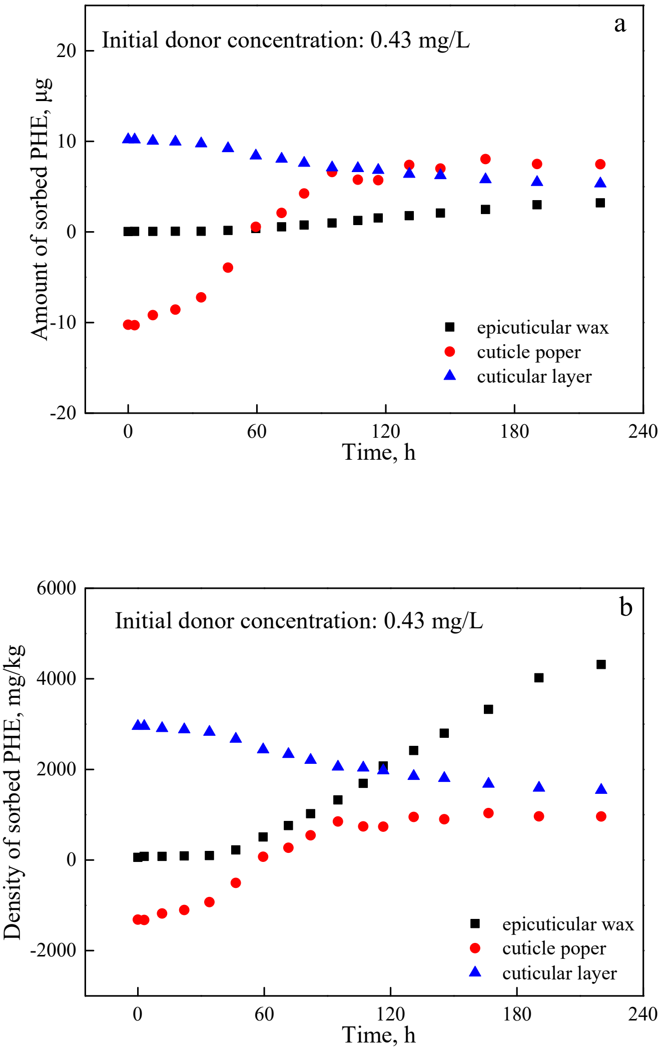

Figure S4. Amount (a) and density (b) of phenanthrene absorbed on epicuticular waxes, cuticle proper and cuticular layer of green pepper fruit cuticular membrane during reverse penetration
